# 

**Published:** 1889-03-02

**Authors:** 


### CONTENTS.

**Woman's Help for Woman** . . . . . . 339

**Literature in Plague Time, III.** . . . . . 340

**The Breakdown of the Hospital System** . . . . . 341

**The Doctor's Pulpit.**—"All Sorts and Conditions" of Women . . . . . 343

**Medical Aids and Appliances.**—Pathology of Sea sickness—Teaching the Deaf and Dumb—Swedish Mechanical Exercise—Defective Articulation . . . . . 344

**A Colonial Congress** . . . . . . . 345

**Notes and News** . . . . . . . 346

**Everybody's Page** . . . . . . . 348

**Annotations.** — Modern Neros and Their Fiddles. — Child Suicides — Baby-farming Superseded . . . . 349

**Insomnia.** By Sir WILLIAM MOORE, K.C.I.E., late Surgeon-General with the Government of Bombay . . . . 350

**False Impressions.** By CAROLINE FOTHERGILL, Author of "An Enthusiast," "A Voice in the Wilderness," etc. . . 351

**An Old Army Surgeon** . . . . . . 352

**Glances at the Insane in Other Lands.** New Brunswick 353

**Scraps and Gleanings. Amusements and Relaxation** 354

### EXTRA SUPPLEMENT—THE NURSING MIRROR.

**En Passant.**—The York Home—American Notes—Jewish Nurses—Nursing in South London—Combination of Private Nurses—Birmingham Nurses—The Interviewer Again . lxxxv

**Nurses on Their Travels.**—A Letter from Madeira . lxxxvi

**Starting a District Nurse** . . . . . lxxxvi

**Notes from Australia** . . . . . . lxxxvii

**Everybody's Opinion.**—Certificates—The Combination of Private Nurses.—Nurses as Stewardesses . . . lxxxvii

**The Nurses' Bookshelf. Lectures** . . . . lxxxviii

**Appointments, Vacancies, Notes and Queries** lxxxviii

